# Hepatitis B Immunization in Health Care Workers: Needs and Opportunities

**DOI:** 10.5812/kowsar.1735143X.705

**Published:** 2011-08-01

**Authors:** Giuseppe La Torre, Rosella Saulle, Brigid Unim

**Affiliations:** 1 Department of Public Health and Infectious Diseases, Sapienza University of Rome, Rome, Italy

**Keywords:** Hepatitis B virus, Vaccination, Antibody

Dear Editor,

The aim of the study carried out by Alavian et al. (2011) is to determine anti-HBs antibody titer in dental health providers and to investigate the possible correlation between the level of immunity and a number of relevant  factors [1]. Hepatitis B virus (HBV) is still the major cause of infection in most parts of the world and therefore is a serious global public health problem. About 2 billion people worldwide have been infected with the virus, and about 350 million live with chronic infection [2]. HBV is transmitted primarily through blood and sexual routes, similar to HIV and hepatitis C virus (HCV) [3][4][5]. Furthermore, HBV is an occupational hazard for health workers [2], who might be discouraged to work for infected patients. According to the recommendations of the Word Health Organization (WHO), all infants should receive their first dose of the hepatitis B vaccine immediately after birth, preferably within 24 hours. This is crucial not only in areas in which hepatitis B is highly prevalent, but also important in intermediate- and low-prevalent areas. Delivery of hepatitis B vaccine within 24 hours of birth should be a performance measure in all immunization programmes. The necessity to vaccinate older age groups, including adolescents and adults, is determined by the baseline epidemiology of HBV infection in the country [4]. The disease has been preventable with a safe and effective vaccine since 1982 [2]. As of 2008, hepatitis B vaccine has been incorporated in 177 countries’ national infant immunization programs, and about 69% of the 2008 birth cohort received all three doses of the vaccine [6].

The WHO/UNICEF coverage estimation between 1980 and 2009 indicates that immunization coverage with all three doses of HBV vaccine in infants is in the range of 50 to 79% in Iran [7]. The study carried out by Alavian et al. states that over 35% of the population is exposed to HBV and approximately 2.5% are chronic carriers in Iran [1]. A free vaccination against HBV is available in health care offices in Iran, but, to date, no mandatory vaccination program against HBV has been established for dentists. Furthermore, there are no documented data of infection rates in dentists and dental staff in Iran [8]; in addition, there is no mandatory vaccination program against HBV for dentists in the country [1]. In this survey, only those who had received at least two doses of the HBV vaccine were included. The assessment of anti-HBs antibody titer in vaccinated dental students, professors, clinical assistants, and nonclinical staff in Faculty of Dentistry indicated a statistically significant association between antibody titeration and age, occupation, smoking, complete vaccination series, and time of the last vaccination. No significant association was observed between gender and anti-HBs antibody titeration, and the rate of immunity was almost 70%, whereas approximately 18% were relatively immune and all others were nonimmune [1]. In the multiple regression analysis, only age and time of the last vaccination were found to be independent predictors of immune system response (P < .001 for both variables). The highest immune response was found among dental students (20-29 y) in comparison to professors and other staff [1]. As a result, vaccination of more than 5 years, an incomplete vaccination, and older age have negative influences on immune response. This result might be a product of the strong emphasis on importance of vaccination in dental students’ educational curriculum [1]. The WHO position paper puts great emphasis on the introduction of immunization programs worldwide: it strongly recommends that all regions and associated countries develop goals for hepatitis B control according to their epidemiological situation [7].

In Italy the anti-HBV universal vaccination was introduced in 1991 for newborn and 12-year-old children, and at the beginning of the 20th century, the prevalence of HBV infection was under 2%. From the beginning of the compulsory vaccination campaign in 1991, there was a downfall in HBV incidence with a reduction of 40% from 1988–91 to 1991–99. The reduction rate was 66% among 0- to 14-year-old children and 59% among 15- to 24-yearold individuals [9]. Health care workers are at risk of blood-borne, airborne, and droplet-spread transmission of infectious agents due to frequent and often intensive occupational exposures, which include percutaneous injury, contact with mucous membranes, or nonintact skin with blood or other potentially infectious bodily fluids. Health care workers may also act as potential paths of nosocomial transmission of several infections to patients and other close contacts [10]. In the study by Alvian et al. we noticed that 13% of health care workers were nonimmune, whereas 17.8% were partially immune; in other words, almost one third of the samples were exposed to HBV infection. It has been clearly recognized and documented in the literature that the usage of specific standard precautions and protective equipment is designed to decrease the risk of acquiring or transmitting occupational infections in health care settings. In particular, the most efficient methods of preventing several hospital-acquired infections and resulting morbidity and mortality is through the usage of preexposure immunization [10]. Vaccination of health care workers results in improved patient and employee safety and, therefore, immunizations are strongly recommended against several diseases for health care personnel [11][12]. In conclusion, these results suggest the need for a national vaccination program to improve immunization coverage among dental health care workers in Iran.
